# Predicting wait time for pediatric kidney transplant: a novel index

**DOI:** 10.1007/s00467-023-06232-1

**Published:** 2024-01-12

**Authors:** Alexandra Alvarez, Ashley Montgomery, Nhu Thao Nguyen Galván, Eileen D. Brewer, Abbas Rana

**Affiliations:** 1 Office of Student Affairs, Baylor College of Medicine, 1 Baylor Plaza, Houston, TX 77030 USA; 2 Division of Abdominal Transplantation, Michael E. DeBakey Department of Surgery, Baylor College of Medicine, Houston, TX USA; 3 Division of Pediatric Nephrology, Department of Pediatrics, Baylor College of Medicine, Houston, TX USA

**Keywords:** Kidney transplantation, Pediatrics, Wait time, UNOS, OPTN

## Abstract

**Background:**

Over one thousand pediatric kidney transplant candidates are added to the waitlist annually, yet the prospective time spent waiting is unknown for many. Our study fills this gap by identifying variables that impact waitlist time and by creating an index to predict the likelihood of a pediatric candidate receiving a transplant within 1 year of listing. This index could be used to guide patient management by giving clinicians a potential timeline for each candidate’s listing based on a unique combination of risk factors.

**Methods:**

A retrospective analysis of 3757 pediatric kidney transplant candidates from the 2014 to 2020 OPTN/UNOS database was performed. The data was randomly divided into a training set, comprising two-thirds of the data, and a testing set, comprising one-third of the data. From the training set, univariable and multivariable logistic regressions were used to identify significant predictive factors affecting wait times. A predictive index was created using variables significant in the multivariable analysis. The index’s ability to predict likelihood of transplantation within 1 year of listing was validated using ROC analysis on the training set. Validation of the index using ROC analysis was repeated on the testing set.

**Results:**

A total of 10 variables were found to be significant. The five most significant variables include the following: blood group, B (OR 0.65); dialysis status (OR 3.67); kidney disease etiology, SLE (OR 0.38); and OPTN region, 5 (OR 0.54) and 6 (OR 0.46). ROC analysis of the index on the training set yielded a c-statistic of 0.71. ROC analysis of the index on the testing set yielded a c-statistic of 0.68.

**Conclusions:**

This index is a modest prognostic model to assess time to pediatric kidney transplantation. It is intended as a supplementary tool to guide patient management by providing clinicians with an individualized prospective timeline for each candidate. Early identification of candidates with potential for prolonged waiting times may help encourage more living donation including paired donation chains.

**Graphical Abstract:**

**Figure:**
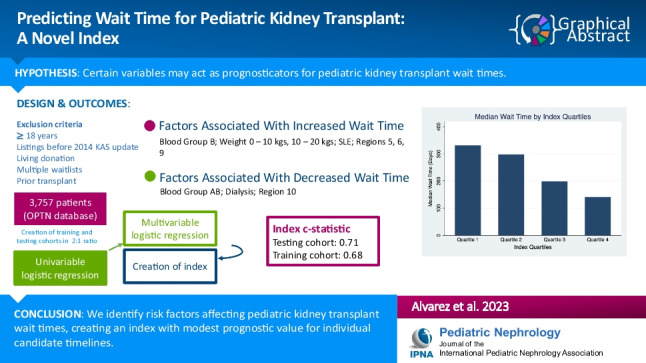
A higher resolution version of the Graphical abstract is available as Supplementary information

**Supplementary information:**

The online version contains supplementary material available at 10.1007/s00467-023-06232-1.

## Background

Kidney allografts remain a scarce resource despite the increasing demand for kidneys over the last decade [1–3]. To create a more equitable system for organ allocation based on this shortage, the Organ Procurement and Transplantation Network (OPTN), operating under the United Network for Organ Sharing (UNOS), routinely updates their kidney allocation system (KAS) to increase accessibility to candidates who are traditionally less likely to receive an organ offer (e.g., highly sensitized patients, racial minorities) [4, 5]. While the OPTN has updated the KAS with moderate success in decreasing racial allocation disparities [4, 5], issues related to allograft availability remain for both adult and pediatric recipients leading to increased waitlist times [1]. For pediatric patients listed in 2016, 76.2% underwent transplant within 3 years; however, 14.2% were still waiting [1]. Additionally, 20% of the kidneys available for transplant were discarded per the 2020 OPTN/SRTR annual report, reaching an all-time high for kidney non-utilization and contributing to the scarce supply of kidney organs [2].

Currently, most pediatric kidney transplantation research focuses on post-transplant outcomes, donor availability, the effects of the 2014 and 2021 KAS updates, and racial disparities. Literature addressing specific factors impacting waitlist outcomes in adults shows that multiple comorbidities, minority status, prolonged time on dialysis, and frequent hospitalizations lead to higher mortality and delayed transplantation among waitlisted candidates [6–12]. Limited literature is available about pediatric candidates less than 18 years old. Our study, the first of its kind, seeks to better understand what factors contribute to pediatric kidney waitlist times and then use these factors to create a novel index which clinicians may use to better estimate the likelihood of deceased donor kidney transplantation within 1 year of listing.

## Methods

### Study population

We performed a retrospective, multi-center, longitudinal analysis of de-identified data from 3757 patients provided by the United Network for Organ Sharing (UNOS) database from 2014 to 2020. Exclusion criteria included patients who were 18 years of age or older, who had a previous transplant of any kind, who were on an additional waitlist for another organ, who had a concurrent transplant, who had a live donor transplant, and who were listed prior to the implementation of the 2014 KAS allocation policy change. Patients who were removed from the waitlist for reasons other than transplantation were censored as our only measured outcome was receipt of transplant within 1 year of listing.

### Statistical analysis

Statistical analysis was performed using Stata 17.0 (Stata Corp). The data was randomly divided into a training set, comprising of two-thirds of the data, and a testing set comprising one-third of the data. A univariable and multivariable logistic regression analysis was performed on the training set to detect factors that were significant (*p*-value < 0.05) in predicting time spent on the waitlist. The primary outcome for this analysis was receipt of a transplant within 1 year of candidate listing. Only variables that were significant (*p*-value < 0.05) in the univariable analysis were included in the multivariable analysis.

### Missing data

Variables with incomplete observations underwent multiple imputation via predictive mean matching with fifth nearest neighbor discriminant analysis. Five iterations of imputation were performed for each variable with missing data. Variables that underwent multiple imputation included the following: BMI-for-age and height-for-age. Percent completion of the data before and after imputation is shown in Table SM1 in Supplementary Materials.

### Variables

The variables considered for this study are shown in Table 1. Variables were recorded at the time of listing and taken directly from the UNOS/OPTN database. Continuous variables were categorized into clinically relevant groupings with reference ranges determined using clinical judgement. Reference variables can be seen in Table 2. BMI-for-age and height-for-age were used in lieu of BMI. Due to the different sizes of growing pediatric patients, BMI that is not adjusted for age has poor predictive value in the pediatric population [13]. Both of the above anthropometric variables are validated measurements for ages 0–19 years, per the World Health Organization (WHO); the measurements are standardized based on *z*-scores set by the WHO 2007 child growth standards [14]. These variables were created using the “zanthro” package provided by Stata, which utilizes the WHO 2007 growth standards [15].

**Table 1:** Demographics of study population

Variable	*N*		*N*%		Median days on waitlist		Standard deviation	
	Train	Test	Train	Test	Train	Test	Train	Test
*Age*								
≤ 1 year	144	68	3.83	1.81	337	279	404	449
> 1 and ≤ 2 years	125	66	3.33	1.76	248	262	462	440
> 2 and ≤ 5 years	285	109	7.59	2.90	291	290	387	408
> 5 and ≤ 10 years	390	222	10.38	5.91	233	203	393	385
> 10 and ≤ 14 years	610	275	16.24	7.32	212	209	367	395
> 14 and ≤ 18 years	951	512	25.31	13.63	225	248	402	410
*Blood group*								
Group A	770	357	20.50	9.50	226	189	382	408
Group B	307	174	8.17	4.63	293	356	400	407
Group O	1338	674	35.61	17.94	228	256	403	406
Group AB	80	44	2.13	1.17	158	192	334	318
*BMI-for-age (BA)*								
*z*-score_BA_ ≤ − 3.0	13	15	0.35	0.40	215	145	376	707
*z*-score_BA_ > − 3.0 and ≤ − 2.0	70	42	1.86	1.12	205	300	398	451
*z*-score_BA_ > − 2.0 and < 1.0	1471	760	39.15	20.23	217	220	398	393
*z*-score_BA_ ≥ 1.0 and < 2.0	478	220	12.72	5.86	276	250	405	365
*z*-score_BA_ ≥ 2.0 and < 3.0	330	146	8.78	3.89	247	267	366	449
*z*-score_BA_ ≥ 3.0	143	69	3.81	1.84	300	313	392	450
*Height-for-age (HA)*								
*z*-score_HA_ ≤ − 3.0	233	118	6.20	3.14	245	253	404	431
*z*-score_HA_ > − 3.0 and ≤ − 2.0	341	166	9.08	4.42	219	237	411	384
*z*-score_HA_ > − 2.0 and < 3.0	1886	945	50.20	25.15	230	237	389	406
*z*-score_HA_ ≥ 3.0	45	23	1.20	0.61	399	293	446	463
*Dialysis status*								
On dialysis	1455	763	38.73	20.31	189	202	327	344
Off dialysis	1050	489	27.95	13.02	307	307	455	469
*Race/ethnicity*								
White	972	491	25.87	13.07	219	213	376	402
African American	519	280	13.81	7.45	228	219	395	443
Hispanic	765	383	20.36	10.19	234	269	417	382
Asian	130	55	3.46	1.46	362	346	446	439
Native American	32	9	0.85	0.24	325	301	340	440
Native Pacific Islander	18	9	0.48	0.24	252	501	336	372
Multiracial	69	25	1.84	0.67	190	241	277	388
*cPRA values*								
cPRA = 0	2315	1137	61.62	30.26	231	238	393	409
cPRA > 0 and < 50	147	83	3.89	2.21	217	172	419	353
cPRA ≥ 50	44	32	1.17	0.85	311	469	378	402
*Weight (kg)*								
≥ 0 and < 10 kg	98	47	2.61	1.25	330	426	466	489
≥ 10 and < 20 kg	525	248	13.97	6.60	276	247	391	385
≥ 20 and < 30 kg	285	154	7.59	4.10	203	215	391	408
≥ 30 and < 40 kg	324	171	8.62	4.55	227	209	387	389
≥ 40 and < 50 kg	384	177	10.22	4.71	202	205	391	395
≥ 50 kg	889	455	23.66	12.11	234	245	391	415
*Kidney disease etiology*								
Acquired defect	16	10	0.43	0.27	433	224	418	536
Amyloidosis	0	1	0.00	0.03	––	108	––	––
BK virus nephropathy	2	1	0.05	0.03	222	241	161	––
Cancer-associated nephropathy	17	5	0.45	0.13	220	162	505	552
Diabetes mellitus I, II	4	1	0.11	0.03	145	360	297	––
Drug-induced nephropathy	8	9	0.21	0.24	365	613	299	527
Heart failure/hypertension	20	9	0.53	0.24	173	311	192	548
HIV-associated nephropathy	2	2	0.05	0.05	55	260	18	93
Hypoplasia/aplasia/dysplasia	1266	614	33.70	16.34	257	267	418	427
Immune complex-associated nephropathy	61	19	1.62	0.51	212	157	425	198
Ischemic/septic nephropathy	131	71	3.49	1.89	248	246	275	406
MELAS	1	0	0.03	0.00	923	––	––	––
Metabolic pathway disorders	10	4	0.27	0.11	414	231	506	220
Nephritic/nephrotic syndromes	492	245	13.10	6.52	205	203	333	329
Nephrocalcinosis	10	3	0.27	0.08	131	145	257	491
Neurogenic bladder	24	13	0.64	0.35	270	577	495	476
Prematurity-associated nephropathy	1	3	0.03	0.08	749	939	––	547
SLE	67	28	1.78	0.75	314	268	453	389
Tubular dysfunction	12	5	0.32	0.13	542	418	336	310
Unspecified autoimmune disease	2	0	0.05	0.00	425	––	70	––
Unspecified familial kidney disease	7	2	0.19	0.05	197	231	465	154
Unspecified glomerulonephritis	50	27	1.33	0.72	243	294	222	453
Unspecified interstitial nephritis	10	4	0.27	0.11	411	542	537	835
Vasculitis	106	94	2.82	2.50	195	158	289	279
Unknown etiology	186	82	4.95	2.18	166	234	402	461
*Insurance type*								
Private insurance	780	382	20.76	10.17	240	240	408	405
Public insurance	1593	791	42.40	21.05	233	242	383	401
Other payment source	132	79	3.51	2.10	175	189	455	467
*OPTN region*								
Region 1	95	61	2.53	1.62	245	270	368	403
Region 2	249	136	6.63	3.62	246	242	413	432
Region 3	331	176	8.81	4.68	179	157	340	364
Region 4	297	135	7.91	3.59	160	195	296	374
Region 5	524	274	13.95	7.29	332	388	473	449
Region 6	118	41	3.14	1.09	351	341	352	354
Region 7	155	77	4.13	2.05	266	266	410	431
Region 8	177	68	4.71	1.81	192	138	273	196
Region 9	175	85	4.66	2.26	357	336	446	432
Region 10	170	87	4.52	2.32	128	174	293	209
Region 11	214	112	5.70	2.98	266	212	379	439
Total sample size = 3757								

Total sample size was 3757. Adolescents aged 14 to 18 years were the largest age group, comprising nearly 39% of the study population, while children under 1 year of age only comprised about 6%. The majority of patients had type O blood, followed by type A, then type B, and lastly type AB, which follows the prevalence of the general population. Both BMI-for-age and height-for-age followed a normal distribution, with the majority of patients falling between *z*-scores − 2 and 1 and − 2 and 3, respectively. These *z*-scores correspond with clinically normal anthropometric measurements. The majority of patients were on dialysis (59%). Additionally, White was the most common race/ethnicity reported (39%), with Hispanic and African American being the other two most reported race/ethnicities. Native Pacific Islander was the least reported race/ethnicity, comprising only 0.72%. Over 90% of patients had a cPRA value of 0. Thirty-six percent of patients weighed over 50 kg, 39% between 20 and 50 kg, and 21% between 10 and 20 kg. The remaining 4% of patients weighed less than 10 kg. The majority of our study population had public insurance (63%), while 31% had private insurance. Finally, the most common region reported was region 5, followed by 3, 4, and 2. The remaining regions were similar in frequency, each comprising around 4–6% of our study population

**Table 2:** **Univariable logistic regression**. This table displays factors that were significant in the univariable analysis, as well as the reference group for all variables, where applicable. Age shows a bimodal distribution of significance with children less than one year old and children between 10–14 years being independently associated with either decreased or increased likelihood to transplantation. Notably, dialysis status is highly significant with a very large odds ratio of 3.37. Multiple regions are also significant, particularly region 9 which is associated with a 51% decrease in likelihood to transplantation within one year of listing. Blood groups B and AB, lower weight values, kidney hypoplasia/aplasia/dysplasia, nephritic/nephrotic syndromes, SLE, vasculitis, and public insurance are also factors significant in the univariable analysis. BMI-for-age, race/ethnicity (except Asian), and cPRA values are not significant. A full table showing the univariable analysis results for all variables can be seen in Supplemental Materials

Variable	Odds ratio	*p*-value	95% confidence interval	
*Age*				
≤ 1 year	0.56	0.00	0.39	0.81
> 2 and ≤ 5 years	*Reference*			
> 10 and ≤ 14 years	1.26	0.01	1.05	1.51
*Blood group*				
Group B	0.73	0.01	0.57	0.93
Group O	*Reference*			
Group AB	1.86	0.01	1.18	2.92
*BMI-for-age (BA)*				
*z*-score_BA_ > − 2.0 and < 1.0	*Reference*			
*Height-for-age (HA)*				
*z*-score_HA_ > − 2.0 and < 3.0	*Reference*			
*z*-score_HA_ ≥ 3.0	0.27	0.00	0.13	0.58
*Dialysis status*				
On dialysis	3.37	0.00	2.84	3.99
Off dialysis	*Reference*			
*Race/ethnicity*				
White	*Reference*			
Asian	0.66	0.03	0.46	0.96
*cPRA values*				
cPRA = 0	*Reference*			
*Weight (kg)*				
≥ 0 and < 10 kg	0.40	0.00	0.25	0.64
≥ 10 and < 20 kg	0.82	0.04	0.67	0.99
≥ 20 and < 30 kg	*Reference*			
*Kidney disease etiology*				
Hypoplasia/aplasia/dysplasia	0.82	0.01	0.70	0.96
Nephritic/nephrotic syndromes	1.43	0.00	1.17	1.74
SLE	0.54	0.02	0.32	0.91
Vasculitis	1.64	0.01	1.11	2.42
Unknown etiology	*Reference*			
*Insurance type*				
Public insurance	1.28	0.00	1.09	1.51
Other payment source	*Reference*			
*OPTN region*				
Region 2	0.73	0.02	0.55	0.95
Region 3	1.60	0.00	1.27	2.02
Region 4	1.93	0.00	1.51	2.46
Region 5	0.51	0.00	0.41	0.62
Region 6	0.59	0.01	0.40	0.88
Region 8	1.68	0.00	1.24	2.29
Region 9	0.49	0.00	0.35	0.68
Region 10	1.86	0.00	1.36	2.55

Due to the heterogeneity of our population's primary diagnoses and these diagnoses being coded using non-standardized language in the UNOS database, a new variable was created to provide an accurate reflection of the prevalence of the diseases in our cohort. Each data point was manually selected and placed into appropriate groupings of matching or similar pathologies so that each etiology was appropriately coded for. Each diagnosis was then turned into its own dummy variable for analysis. The specific diagnoses included in each etiology grouping can be seen in Table SM2 in the Supplementary Materials.

The following characteristics were included in the univariable analysis: age (years); blood group, A, B, O, and AB; BMI-for-age; height-for-age; dialysis status; race/ethnicity, white, African American, Hispanic, Asian-American, Native American, Native Pacific Islander, and multiracial; cPRA value; weight (kilograms); kidney disease etiology, acquired kidney defect, amyloidosis, BK virus nephropathy, cancer-associated nephropathy, diabetes, drug-induced nephropathy, heart failure/hypertension, HIV-associated nephropathy, hypoplasia/aplasia/dysplasia, immune complex-associated nephropathy, ischemic/septic nephropathy, MELAS, metabolic pathway disorders, nephrotic/nephritic syndromes, nephrocalcinosis, neurogenic bladder, prematurity-associated nephropathy, SLE, tubular dysfunction, unspecified autoimmune disease, unspecified familial kidney disease, unspecified glomerulonephritis, unspecified interstitial nephritis, vasculitis, and unknown etiology; insurance type, private and public; and UNOS region, 1, 2, 3, 4, 5, 6, 7, 8, 9, 10, and 11 (see Supplementary Materials for OPTN region map [16]).

### Risk index

Only variables that were significant in the multivariable analysis were used to create the risk index. The development of the index was modeled after the liver transplant length of stay index by Rana et al. [17]. The presence or absence of each significant variable, coded as either one or zero, respectively, was multiplied by the logarithmic function of their corresponding odds ratio. The exponential function of the sum of these values yielded an index score. The index was validated for its ability to predict transplantation within 1 year of listing using ROC analysis on both the training and testing sets.

## Results

### Demographics

Total sample size following exclusionary criteria was 3757 patients. Our study cohort was composed primarily of adolescent patients between the ages of 14 and 18 years old (39%) with a progressive decrease in frequency in lower age groups. Only 6% of our study population was 1 year old or younger. Group O blood type was the most common blood group at 54%, followed by group A at 31%. Group B and group AB comprised 13% and 3% of the study cohort, respectively. Growth parameter BMI-for-age had a normal distribution with most patients (59%) falling between *z*-scores − 2.0 and 1.0. Height-for-age had a normal distribution with a slight positive skew. The majority of patients (75%) fell between *z*-scores − 2.0 and 3.0. Thirty-six percent of our study population weighed 50 kg or more, while 21% of patients weighed between 10 and 20 kg. Only 4% weighed less than 10 kg. Over half of our patients were on dialysis (59%). Ninety-one percent of our cohort had a cPRA value of zero. Regarding race, 39% of our study was White, 31% Hispanic, 21% African American, and 5% Asian. The remaining 4% were Native American, Native Pacific Islander, or multiracial. The four most common kidney disease etiologies were kidney hypoplasia, dysplasia, and aplasia (50%); nephrotic and nephritic syndromes (20%); ischemic or septic nephropathy (5%); and vasculitis (5%). Unknown etiologies comprised 7% of the cohort. Almost two-thirds of our study population had some form of public insurance (63%), approximately one-third (31%) had private insurance, and 6% had other payment sources, such as charity or self-pay. Finally, 21% of our study population was located in region 5, while approximately 70% of our population was evenly distributed among regions 2, 3, 4, 7, 8, 9, 10, and 11. Only 8% of our study population was located in regions 1 or 6.

### Univariable and multivariable analysis

Table 2 includes all factors assessed in the univariable analysis. Only variables significant in the univariable analysis were included in the multivariable analysis, shown in Table 3. Variables that were significant and examined in the multivariable analysis include the following: age (years), < 1, > 10, and ≤ 14; blood group, B and AB; height-for-age, *z*-score > 3; dialysis status; race/ethnicity, Asian; weight (kilograms), ≥ 0 and < 10, and ≥ 10 and < 20; kidney disease etiology, hypoplasia/aplasia/dysplasia, nephrotic/nephritic syndromes, SLE, and vasculitis; insurance type, public; and UNOS region, 2, 3, 4, 5, 6, 8, 9, and 10.

**Table 3:** **Multivariable logistic regression results**. This table displays factors that were significant in the multivariable analysis. Blood groups B and AB, dialysis status, weight between 10 and 20 kg, SLE, and regions 5 and 6 were the most significant factors from this analysis. Dialysis status, SLE, and regions 5 and 6 had the most clinically significant odds ratios. Patients on dialysis were over three times more likely to receive a transplant within one year of listing while patients who had SLE had a 62% decreased likelihood for the same event. Patients located in regions 5 or 6 had a 46% and 54% decreased likelihood, respectively. A full table showing the multivariable analysis results for all tested factors can be seen in Supplemental Materials

Variable	Odds ratio	*p*-value	95% confidence interval	
*Blood group*				
Group B	0.65	0.00	0.49	0.84
Group AB	2.07	0.00	1.25	3.40
*Dialysis status*				
On dialysis	3.67	0.00	3.03	4.45
*Weight (kg)*				
≥ 0 and < 10 kg	0.43	0.01	0.23	0.82
≥ 10 and < 20 kg	0.59	0.00	0.47	0.76
*Kidney disease etiology*				
SLE	0.38	0.00	0.21	0.68
*OPTN region*				
Region 5	0.54	0.00	0.41	0.71
Region 6	0.46	0.00	0.30	0.73
Region 9	0.58	0.01	0.39	0.87
Region 10	1.64	0.01	1.13	2.40

Variables that were significant (*p*-value < 0.05) in the multivariable analysis include the following: blood group, B and AB; dialysis status; weight (kilograms), ≥ 0 and < 10, and ≥ 10 and < 20; kidney disease etiology, SLE; and OPTN region, 5, 6, 9, and 10. The most significant predictive factors for likelihood to transplantation within 1 year of listing include the following: blood group, B (OR 0.65, *p*-value = 0.00); dialysis status (OR 3.67, *p*-value = 0.00); kidney disease etiology, SLE (OR 0.38, *p*-value = 0.00); and OPTN region, 5 (OR 0.54, *p*-value = 0.00) and 6 (OR 0.46, *p*-value = 0.00).

### Risk index

Significant variables (*p*-value < 0.05) in the multivariable analysis were used to create the risk index, which can be calculated using the following equation:

$$\mathrm{index}=\exp(\left(-0.19\;\mathrm{if}\;\mathrm{Blood}\;\mathrm{Group}\;\mathrm B\right)+\left(0.31\;\mathrm{if}\;\mathrm{Blood}\;\mathrm{Group}\;\mathrm{AB}\right)+\left(0.57\;\mathrm{if}\;\mathrm{on}\;\mathrm{Dialysis}\right)+\left(-0.36\;\mathrm{if}\;\mathrm{Weight}\;0-10\;\mathrm{kg}\right)+\left(-0.23\;\mathrm{if}\;\mathrm{Weight}\;10-20\;\mathrm{kg}\right)+(-0.42\;\mathrm{if}\;\mathrm{dx}\;\mathrm{of}\;\mathrm{SLE})+\left(-0.27\;\mathrm{if}\;\mathrm{in}\;\mathrm{Region}\;5\right)+\left(-0.33\;\mathrm{if}\;\mathrm{in}\;\mathrm{Region}\;6\right)+\left(-0.24\;\mathrm{if}\;\mathrm{in}\;\mathrm{Region}\;9\right)+\left(0.22\;\mathrm{if}\;\mathrm{in}\;\mathrm{Region}\;10\right))$$

**Fig. 1:**
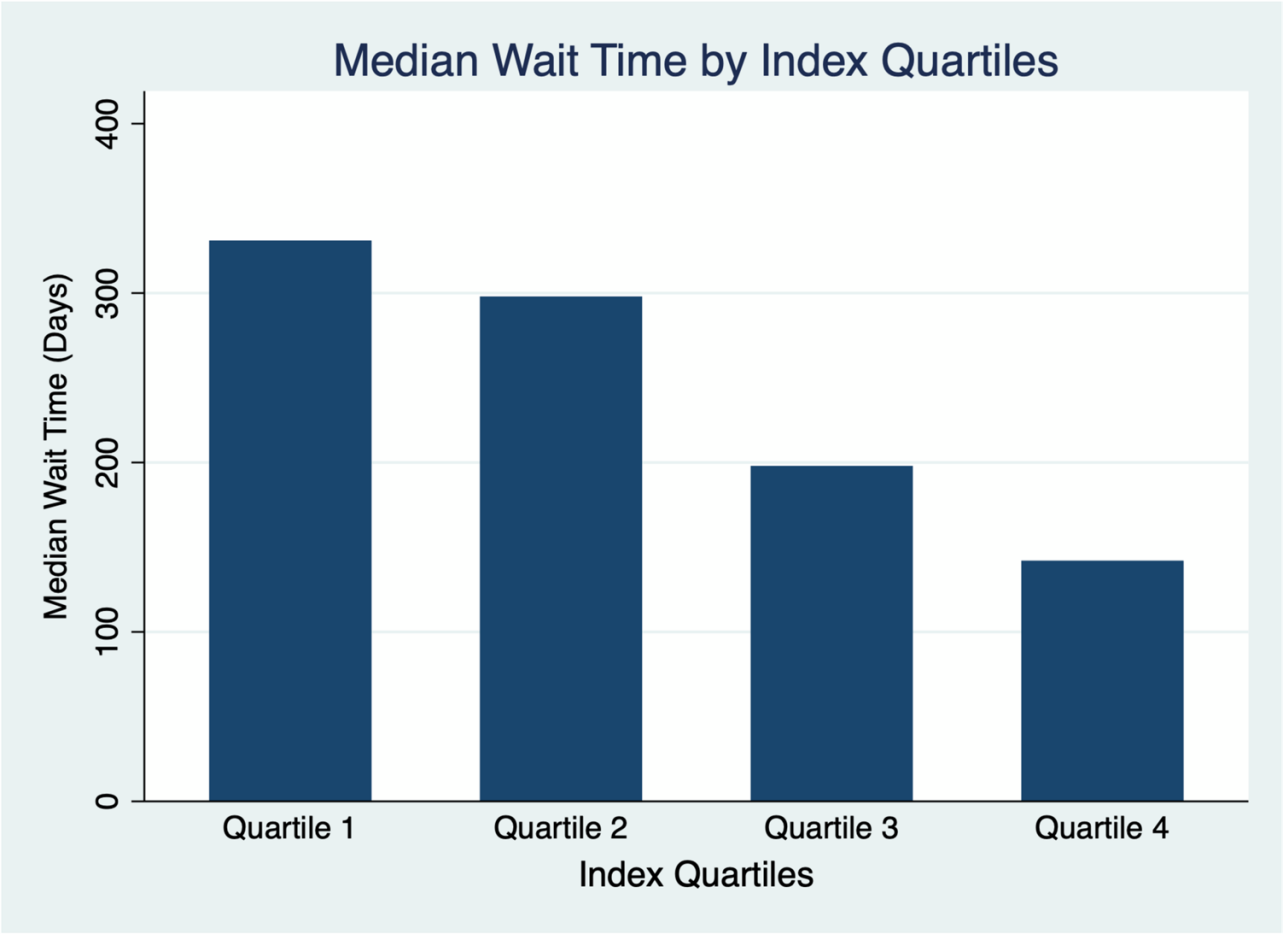
Risk index scores stratified into quartiles and then graphed against their median wait time. As index scores increase, median wait time steadily decreases. Median wait time for those in quartile 4 is less than half of the wait time for patients in quartile 1. The values for each quartile: Quartile 1: ≤ 1.00; Quartile 2: 1.00-1.64; Quartile 3: 1.64-3.67; and Quartile 4: >3.67.

**Fig. 2:**
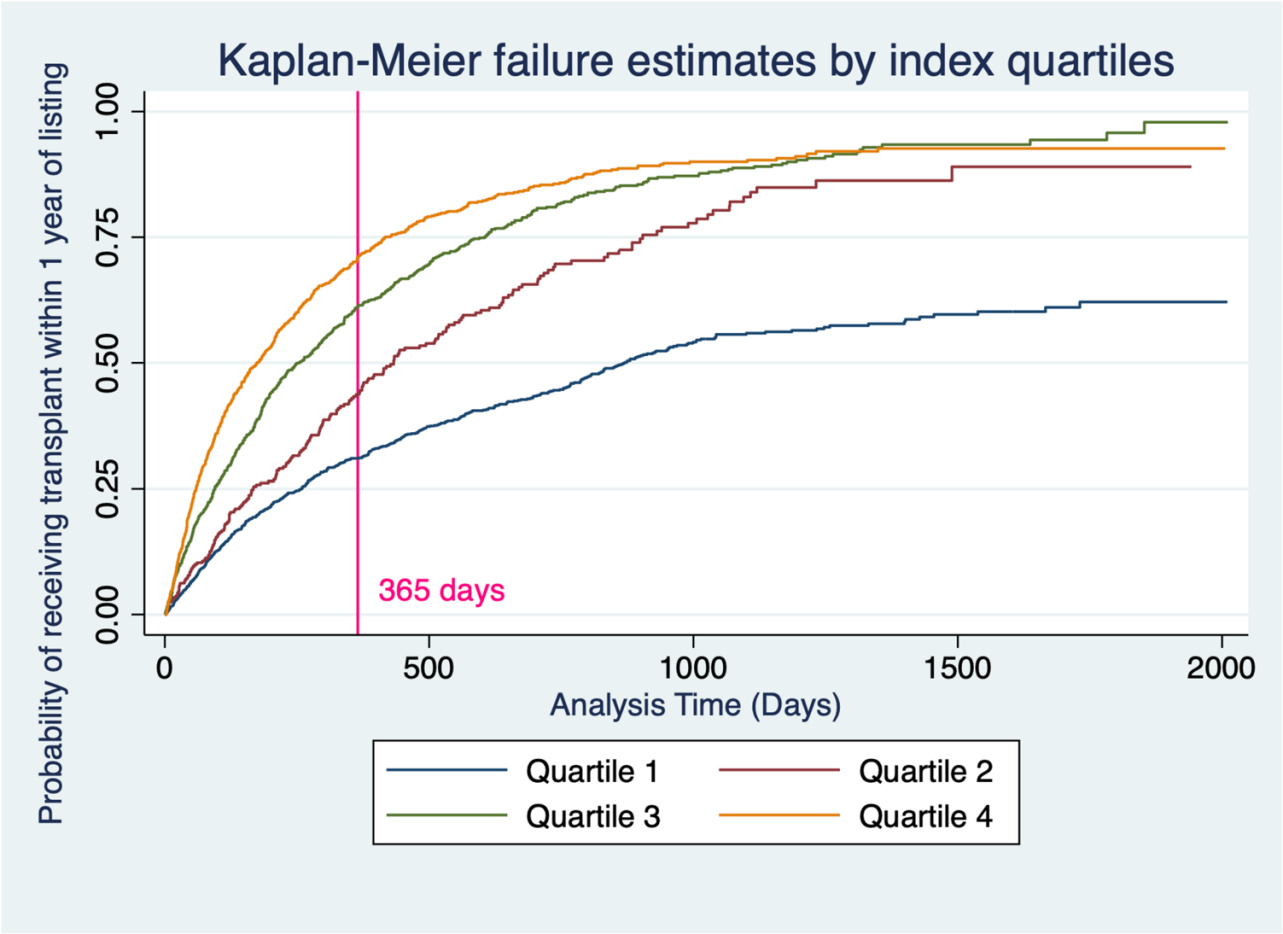
Kaplan-Meier failure estimates for each index quartile displaying likelihood of transplantation within one year of listing. A higher index score is associated with increased event likelihood. A reference line at 365 days is present, representing the one year from listing threshold we designated as our "failure" event. Following the one year mark, index scores in the first quartile start to lag much further behind the other three quartiles with regards to event likelihood. At around 1320 days (approximately 3.5 years) quartile 3 overtakes quartile 4, now having an event likelihood of 93%

**Fig. 3:**
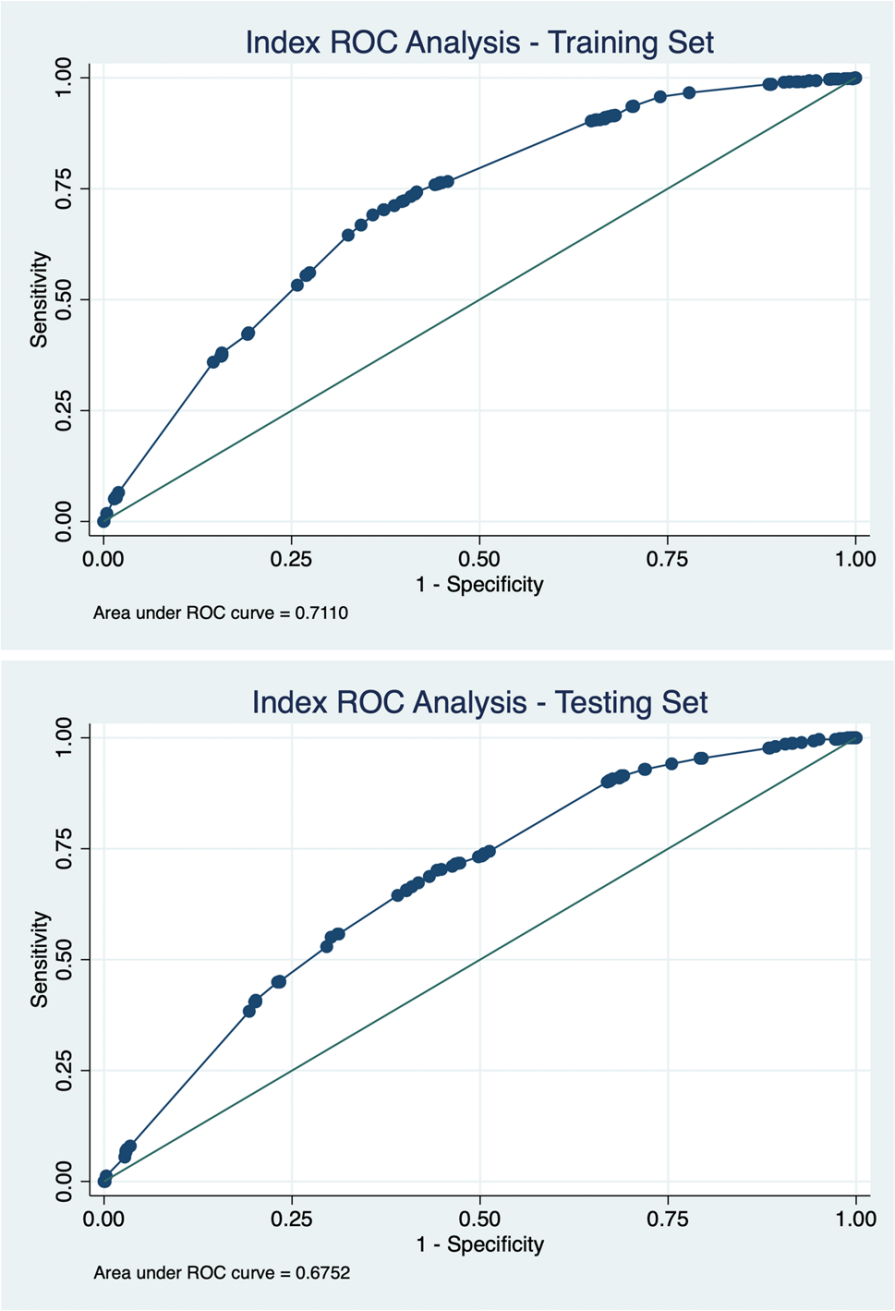
ROC analysis for the index in the training set (**a**) and testing set (**b**). C-statistic values between the two cohorts were fairly concordant at 0.71 and 0.68, respectively

The risk index was stratified into quartiles and then graphed against their median time on the waitlist (Fig. 1). The index quartiles were also used to create a Kaplan–Meier failure estimate curve displaying event likelihood (transplantation within 1 year of listing) over time (Fig. 2). Patients with a lower index score had higher median wait times with those in the bottom tenth percentile having a median wait time of 532 days. Patients with a higher index score had lower median wait times with the top tenth percentile having a median wait time of 142 days. ROC analysis of the index against the training set yielded a c-statistic of 0.71 (Fig. 3a). Examined against the testing set, ROC analysis of the index revealed a c-statistic of 0.68 (Fig. 3b).

## Discussion

The novel pediatric kidney transplant waitlist index developed by this study includes 10 variables that were all determined to be significant (*p*-value < 0.05) in the multivariable analysis. They include the following: blood group, B and AB; dialysis status; weight (kilograms), ≥ 0 and < 10, and ≥ 10 and < 20; kidney disease etiology, SLE; and OPTN region, 5, 6, 9, and 10. ROC analysis of the index against the training and testing sets revealed a c-statistic of 0.71 and 0.68, respectively, indicating that this index is a modest prognosticator of the likelihood of pediatric kidney transplantation within 1 year of listing. A resulting c-statistic of 0.5 indicates random association while a c-statistic of 1.0 indicates a perfect association.

Our study is the first of its kind to investigate multiple factors affecting waitlist times specific to pediatric kidney transplantations. Previous literature analyzing factors impacting waitlist times has been focused on the adult population and identified different factors for adults compared to our findings for pediatrics. The adult studies identified factors such as PRA status, blood type, and age to be significant predictors affecting waitlist time for deceased-donor kidney transplant, and increased comorbidity burden was associated with waitlist mortality [6, 7]. Additionally, hospitalization within the first year of waitlisting was associated with a lower likelihood of receiving a kidney transplant and worse 5-year survival, which progressively decreased with increased hospital length of stay [8]. In adults, factors of age, diabetes, cardiovascular disease, and chronic obstructive pulmonary disease also contributed to increased mortality [8]. Racial disparities were also identified and have been identified in pediatric patients as well [11].

Black and Hispanic patients are both found to have decreased likelihood of receiving a transplant compared to non-Hispanic White patients, with disparities persisting into young adulthood [11, 12, 18]. However, reports by Melanson et al. indicate amelioration of these disparities through the 2014 KAS update [5]. Despite the longstanding history of racial disparities among pediatric and adult kidney transplant patients, our study did not find race to be a significant factor for predicting time to transplantation. One possibility for this finding is that minority patients become transplant candidates at a more advanced stage of disease due to racial bias upstream in their care, preventing them from being preemptively waitlisted. Another possibility is that the KAS update objective to reduce racial disparities in transplantation has been successful. Per the annual OPTN/UNOS reports, discrepancies in rate of transplantation between white and minority patients have continued to decline [2, 19].

Our pediatric waitlist index includes several variables that have been independently reported as predictive risk factors for decreased rates of transplantation. For example, blood group B has been widely reported as being associated with decreased likelihood of organ transplant, with much of the literature suggesting the implementation of ABO incompatible (ABOi) transplants to compensate for this disproportionate gap in transplants between blood groups [1, 20, 21]. Additionally, geographic variation in access to transplantation has been documented as an independent risk factor for decreased rates of transplantation. As shown by Mathur et al. in 2010, areas with increasing kidney failure incidence have diminished access to the transplant waitlist when compared to patients in low kidney failure incidence areas even after accounting for local donation rates [22]. Areas with moderate to high kidney failure incidence corresponded most with UNOS regions 2, 3, 4, 5, 9, 10, and 11 [22]. Further, research by Reese et al. in 2014 reported similar findings of geographic variation in wait times for pediatric patients with wait times most increased in areas corresponding to regions 2, 5, and 9 [23]. These findings are similar to our study which found regions 5, 6, and 9 to be associated with a decreased likelihood of transplantation. Overall inequities in wait times among OPTN regions have been attributed to inadequate supply of locally procured organs in the face of increasing demand in regions with a high proportion of highly sensitized minority patients [24]. Additionally, the differing criteria for organ acceptance versus rejection for each individual transplant center have been suggested as a contributing factor toward regional variation in wait times [25].

Such center-specific practices must be considered as there are no specific criteria set by the KAS that patients need to meet to be waitlisted [26]. Parameters such as GFR and dialysis status exist in the KAS to aid in calculation of wait times for candidates (i.e., pediatric patients’ time on dialysis prior to listing will count toward their wait time; patients with longer wait times are considered higher priority). Additionally, listing protocol for each center may be further varied based on the patient’s disease. For example, children with congenital malformations may be listed at earlier stages in their disease if it is anticipated they will require a transplant compared to older children with acquired kidney failure, leading to a “prolonged” wait time. King et al. found that variations in wait time among patients of similar characteristics (e.g., disease status, sensitization, comorbidities, age) were smaller compared to the overall variation between centers within specific regions [25]. These findings suggest that center-specific listing and transplant protocols, rather than patient characteristics themselves, have significantly more influence on wait time than previously thought.

Age was not found to be a significant predictor of likelihood of transplantation. This does not align with the previous literature as, historically, patients have a progressive increase in likelihood for transplantation with decreasing age, likely due to receiving higher pediatric priority points and decreased likelihood of sensitization compared to adolescents [27, 28]. One consideration is that younger, smaller candidates pose more difficulties in size-matching donor kidneys, as well as increased risk for thrombosis due to small vessel size [29, 30]. Our findings that candidates weighing less than 20 kg have as much as a 57% decreased likelihood of transplantation within a year of listing would support this notion. However, advances in infant kidney transplantation have allowed for decreased rates of graft failure with improved long-term outcomes, thereby removing the need to prolong wait times while the patient grows to an adequate height and weight [29–31]. Such advances are likely to contribute toward younger age groups not being statistically significant in our study.

Heavier weight groups were not found to be statistically significant in either the univariable or multivariable analysis. Anthropometric variables BMI-for-age and height-for-age were also not found to be significant predictors of likelihood to transplantation. These variables are likely to be impacted by disease severity as kidney failure has a well-known effect on bone mineralization and vertical growth [32–34]. Given the heterogeneity of kidney failure etiology and age of disease presentation, these growth parameters may be a poor predictor of wait time, as shown in our study. Further analysis of our data does not show a correlation between any growth parameters and age.

Interestingly, cPRA was not found to be a significant predictor of likelihood to transplantation in either direction. This does not align with previous findings showing significant increases in the rate of transplantation for highly sensitized patients with elevated cPRA values, an intended result by the 2014 KAS update [35–39]. Previous literature has also shown a resultant significant decline in rate of transplantation for those with lower cPRA values, primarily those with a value of zero percent [38, 39]. Our findings are likely due to priority points being assigned to patients with increasing cPRA values, counterbalanced by the difficulty in finding a donor that has minimal to no reactive antigens [26].

Systemic lupus erythematous was the only kidney disease etiology found to be a significant predictor with patients in our study being 62% less likely to receive a transplant within one year of listing. It has been thought that allowing for disease remission and symptom quiescence via an extended waiting period would prevent post-transplantation complications and allograft loss [41–43]. This practice is a likely contributor to our finding however, evidence that this longer wait time provides any benefit has been mixed, with some studies showing an increased risk of graft failure [42–44]. 

Finally, dialysis status was a significant predictive variable for likelihood to transplantation. Patients who are on dialysis are over three times as likely to receive a transplant within a year of listing compared to their non-dialysis peers. This finding is similar to that of Shelton et al. and is also in line with the KAS protocol, which counts time on dialysis prior to listing towards wait time, which in turn increases listing priority [26, 40].

Our index not only includes significant predictive factors impacting the likelihood of transplantation but may also provide important insights about potential prolonged waitlist times for a given candidate. For example, our index was inversely related to wait time; patients with an index score in the bottom ten percent have a median wait time over twice as long as those in the top ten percent. This important finding is likely to have clinical implications. Our index may provide early identification of candidates at risk for prolonged waitlist time, for whom interventions might be made to lower morbidity while awaiting a donor kidney and to find potential living donors to obviate waitlist time.

Such implications have yet to be applied to countries outside of the United States as this index is based on US population-specific factors. Further research is needed utilizing foreign transplant databases as variables that will be significant may differ due to diverse patient characteristics that are dissimilar to those seen in the US. Patient characteristics such as comorbidities, age of disease presentation, and age of listing may differ due to societal influences, including healthcare accessibility, racial biases in healthcare, and environmental factors contributing to the level of obesity, diabetes, and heart disease in the general population.

## Potential limitations

The UNOS database is subject to inaccurate data input as exemplified by the non-standardized and mistakenly coded diagnoses that necesitated recoding and reorganization of the data. However, the strength of this database lies in its longitudinal nature and massive sample size. Even after excluding data prior to the 2014 KAS update, we were able to maintain a large sample size, thereby increasing the power of our study. An additional limitation includes the very slight model performance mismatch as ROC analysis yielded differing c-statistics between the training and testing sets, although this result is to be expected. C-statistic discordance can be due to under-representative data in either set; however, this was mitigated by sampling randomization when splitting the dataset into the two cohorts.

## Conclusions

This retrospective, multi-center, longitudinal analysis of 3757 pediatric kidney transplant candidates was performed to identify predictive factors impacting time spent on the deceased donor waitlist. A pediatric waitlist index was created after identifying 10 significant candidate factors that influence likelihood of transplantation within 1 year of being placed on the waitlist. Our index had a c-statistic of 0.71 and 0.68 when derived from the training and testing sets, respectively. The most important factors in this index are blood group, dialysis status, kidney disease etiology, and UNOS region. This index is intended to be used as a supplementary tool to help guide clinical management of pediatric transplant candidates. We hope that the modest prognostication provided by our model may empower clinicians and families to reduce waitlist morbidity, find more living donors, and utilize more paired donation chains to optimize long-term pediatric patient outcomes. Further research is needed to optimize this index and further increase its predictive power, as well as to generate an index that is applicable to populations outside of the United States.

### Supplementary information

Below is the link to the electronic supplementary material.

Graphical abstract (PPTX 297 kb)

Supplementary file1 (DOCX 55.5 KB)

## Data Availability

Data utilized in this study were from the United Network for Organ Sharing public database. Statistical analysis utilized Stata 17.0 (Stata Corp).

## Abbreviations

cPRA: Calculated panel reactive antibody
GFR: Glomerular filtration rate
HLA: Human leukocyte antigen
KAS: Kidney allocation system
OPTN: Organ Procurement and Transplantation Network
UNOS: United Network for Organ Sharing

## Declarations

The authors have nothing to disclose. Due to using de-identified patient information, IRB approval was not needed and therefore waived.
