# Correction: Predicting wait time for pediatric kidney transplant: a novel index

**DOI:** 10.1007/s00467-024-06401-w

**Published:** 2024-06-04

**Authors:** Alexandra Alvarez, Ashley Montgomery, Nhu Thao Nguyen Galván, Eileen D. Brewer, Abbas Rana

**Affiliations:** 1 Office of Student Affairs, Baylor College of Medicine, 1 Baylor Plaza, Houston, TX 77030 USA; 2 Division of Abdominal Transplantation, Michael E. DeBakey Department of Surgery, Baylor College of Medicine, Houston, TX USA; 3 Division of Pediatric Nephrology, Department of Pediatrics, Baylor College of Medicine, Houston, TX USA

**Correction: Pediatric Nephrology**

10.1007/s00467-023-06232-1

Within this study, the authors constructed a pediatric waitlist risk index and stratified this index into quartiles. However, in the original manuscript, they did not include the range of values that each quartile spans. This has been added to the caption of Fig. 1.

Please see the previous figure caption here:

Risk index scores stratified into quartiles and then graphed against their median wait time. As index scores increase, median wait time steadily decreases. Median wait time for those in quartile 4 is less than half of the wait time for patients in quartile 1.

The updated figure caption reads:

Risk index scores stratified into quartiles and then graphed against their median wait time. As index scores increase, median wait time steadily decreases. Median wait time for those in quartile 4 is less than half of the wait time for patients in quartile 1. The values for each quartile: Quartile 1: ≤ 1.00; Quartile 2: 1.00–1.64; Quartile 3: 1.64–3.67; and Quartile 4: > 3.67.

The original article has been corrected.

