# Supplementary material for: Predicting wait time for pediatric kidney transplant: a novel index
Source: Pediatr Nephrol. 2024 Jan 12;39(8):2483–93. doi: 10.1007/s00467-023-06232-1 (PMC11199301; doi:10.1007/s00467-023-06232-1)
Supplement: Supplementary file 1 — (PPTX 297 kb) [file 467_2023_6232_MOESM1_ESM.pptx]

## Slide 1
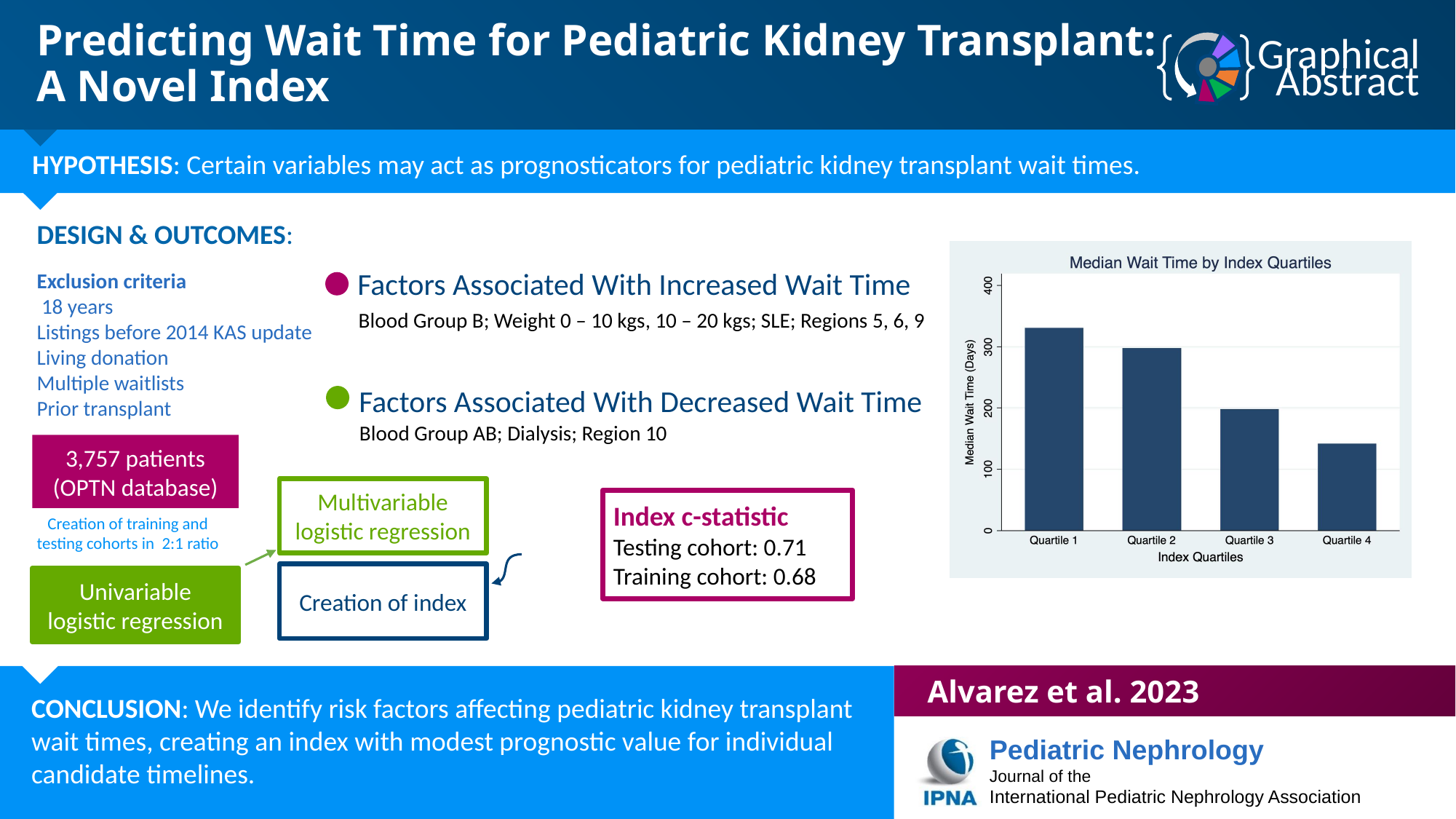

Predicting Wait Time for Pediatric Kidney Transplant:
A Novel Index
HYPOTHESIS: Certain variables may act as prognosticators for pediatric kidney transplant wait times.
DESIGN & OUTCOMES:
Factors Associated With Increased Wait Time
Blood Group B; Weight 0 – 10 kgs, 10 – 20 kgs; SLE; Regions 5, 6, 9
Factors Associated With Decreased Wait Time
Blood Group AB; Dialysis; Region 10
3,757 patients (OPTN database)
Multivariable logistic regression
Index c-statistic
Testing cohort: 0.71
Training cohort: 0.68
Creation of training and testing cohorts in 2:1 ratio
Creation of index
Univariable logistic regression
Alvarez et al. 2023
CONCLUSION: We identify risk factors affecting pediatric kidney transplant wait times, creating an index with modest prognostic value for individual candidate timelines.
