# Supplementary material for: Predicting wait time for pediatric kidney transplant: a novel index
Source: Pediatr Nephrol. 2024 Jan 12;39(8):2483–93. doi: 10.1007/s00467-023-06232-1 (PMC11199301; doi:10.1007/s00467-023-06232-1)
Supplement: Supplementary file 2 — Supplementary file1 (DOCX 55.5 KB) [file 467_2023_6232_MOESM2_ESM.docx]

**Supplementary Figures**

**
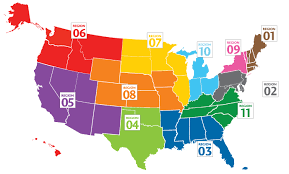
**

**SM Figure 1** Map from OPTN showing the 11 regions in the United States. These geographically-based regions were created for administrative and governance purposes. Previously, these regions were further divided into donor service areas (DSA), each of which contained at least one transplant center and donor hospital. Each DSA was served by one organ procurement organization. The 2021 KSA update has done away with DSAs and have instead replaced them with a 250 nautical mile (NM) fixed circle centered around a donor hospital. Patients located within this circle receive priority points that decrease in value the closer they are to the circle's border. This change was made with the intention of decreasing geographic-based inequities for transplant recipients. [Regions - OPTN. In: Organ Procurement and Transplantation Network. https://optn.transplant.hrsa.gov/about/regions/. Accessed 23 Aug 2022]

**Supplementary Tables**

| **Imputed Variables** | **Number of patients before imputation** | **Percentage complete before imputation** | **Percent complete after imputation** |
| --- | --- | --- | --- |
| Height (cm) | 3,752 | 99% | 100% |
| BMI | 3,739 | 99% | 100% |
| **Total Sample Size = 3,757** | | | |

**SM Table 1** Height and BMI were the only variables that had incomplete data. Though the rate of completion is nearly 100%, we chose to continue with imputation as no other variables had any missing data.

| **Kidney Disease Etiology** | **Included Diagnoses** |
| --- | --- |
| **Acquired Defect** | Acquired obstructive uropathy |
|  | Acquired solitary kidney |
| **Cancer** | Neuroblastoma |
|  | Radiation |
|  | Renal sarcoma |
|  | Wilm’s tumor |
| **Drug Induced Nephropathy** | Chemotherapy induced nephropathy |
|  | Unspecified drug induced nephropathy (excluding teratogens) |
| **Heart Failure/Hypertension** | Heart failure associated nephropathy |
|  | Hypertension associated nephropathy |
| **Hypoplasia/Aplasia/Dysplasia** | ACE/ARB fetopathy |
|  | Adams-oliver syndrome |
|  | Alagille syndrome |
|  | ARPKD |
|  | Bardet biedl syndrome |
|  | Brachio-otorenal syndrome |
|  | CAKUT |
|  | Coach syndrome |
|  | Cogan type oculomotor apraxia |
|  | Cornelia de lange syndrome |
|  | Eagle barrett syndrome |
|  | Fraser syndrome |
|  | IFT140 mutation |
|  | Job syndrome |
|  | Joubert syndrome |
|  | Leber congenital amaurosis |
|  | Modys |
|  | Neurofibromatosis |
|  | NPHP3/4, NPHS2 mutations |
|  | OFD1 deletion |
|  | Orofacial digital syndrome, type1 |
|  | Prune belly syndrome |
|  | PUV/reflux |
|  | Renal coloboma syndrome |
|  | Senior loken syndrome |
|  | Townes-brock syndrome |
|  | Tuberous sclerosis |
|  | Turner syndrome |
|  | VACTERL association |
|  | Wolf hirschorn syndrome |
| **Interstitial Nephritis** | Granulomatous interstitial nephritis |
|  | Interstitial nephritis due to uromodulin mutation |
|  | Unspecified chronic interstitial nephritis |
| **Immune Complex-Associated Nephropathy** | C1Q nephropathy |
|  | C3 glomerulonephritis |
|  | IGM nephropathy |
|  | Unspecified immune complex glomerulonephritis |
| **Ischemic/Septic Nephropathy** | Hemolytic uremic syndrome |
|  | Perinatal hypoxic event/renal infarct |
|  | Renal infarct due to trauma/hemorrhage |
|  | Renal vein thrombosis |
|  | Sepsis |
|  | Sickle cell anemia |
|  | Unspecified hypoxic event/renal infarct |
|  | Unspecified thrombotic microangiopathy |
| **Metabolic Pathway Disorders** | Cytochrome C oxidase deficiency |
|  | Glycogen storage disease |
|  | Leukodystrophy |
|  | Methylmalonic acidemia |
|  | Mitochondrial Myopathy |
|  | Moyamoya |
|  | Nitric oxide synthase polymorphism |
|  | RMNDI1 mutation |
| **Nephritic/Nephrotic Syndromes** | Alport’s syndrome |
|  | Denys-drash syndrome |
|  | Dyskeratosis congenita |
|  | Finish type nephrotic syndrome |
|  | Focal segmental glomerulosclerosis |
|  | Laminin mutation |
|  | Membranoproliferative glomerulonephritis |
|  | Minimal change disease |
|  | Paracellin mutation |
|  | Post-strep glomerulonephritis |
|  | Rapidly progressive glomerulonephritis |
|  | Schimke immuno-osseous dysplasia |
|  | Steroid-resistant nephrotic syndrome |
|  | Unspecified congenital nephrotic syndrome |
|  | Unspecified glomerulosclerosis |
| **Neurogenic Bladder** | Hinman syndrome/non-neurogenic neurogenic bladder |
|  | Spina bifida |
|  | Teratoma |
|  | Unspecified neurogenic bladder |
| **Tubular Dysfunction** | Barter syndrome |
|  | Dent’s disease |
|  | Fanconi syndrome |
|  | Jeune syndrome |
|  | Lowe’s syndrome |
|  | COX10 mutation |
| **Vasculidites** | Anti-GBM |
|  | Granulomatosis with polyangitis |
|  | IgA nephropathy/Henoch Schonlein Purpura |
|  | Microscopic polyangitis |
|  | Polyarteritis nodosa |
|  | Takayasu vasculitis |
|  | Unspecified ANCA vasculitis |
|  | Unspecified vasculitis |
| **Unknown** | AKI |
|  | CKD |
|  | Unknown |

**SM Table 2 Kidney disease etiology variables and their included pathology.** In reviewing the UNOS database diagnosis variables, many pathologies were coded as “999,” meaning other, and subsequently typed into a string variable using non-standardized language. Since this did not provide an accurate reflection of the prevalence of these diseases in our cohort, we manually combed through the data and recoded each variable into diagnostic groupings based on clinical judgement. This table represents the disease etiology variables that comprise multiple individual diagnoses. Diagnoses that fit in multiple groups (e.g. IgA nephropathy being both a vasculitis, as well as an immune complex-based nephropathy) were ultimately placed using clinical judgement.

| **Variable** | **Odds Ratio** | **Standard Error** | **z-score** | **P-value** | **95% Confidence Interval** | |
| --- | --- | --- | --- | --- | --- | --- |
| ***Age*** | | | | | | |
| $\leq$ 1 year | 0.56 | 0.10 | -3.12 | 0.00 | 0.39 | 0.81 |
| > 1 and $\leq$ 2 years | 0.71 | 0.13 | -1.82 | 0.07 | 0.49 | 1.02 |
| > 2 and $\leq$ 5 years | *Reference* | | | | | |
| > 5 and $\leq$ 10 years | 1.23 | 0.14 | 1.86 | 0.06 | 0.99 | 1.52 |
| > 10 and $\leq$ 14 years | 1.26 | 0.12 | 2.45 | 0.01 | 1.05 | 1.51 |
| > 14 and $\leq$ 18 years | 1.02 | 0.08 | 0.28 | 0.78 | 0.87 | 1.20 |
| ***Blood Group*** | | | | | | |
| Group A | 10.98 | 0.09 | -0.27 | 0.79 | 0.82 | 1.16 |
| Group B | 0.73 | 0.09 | -2.55 | 0.01 | 0.57 | 0.93 |
| Group O | *Reference* | | | | | |
| Group AB | 1.86 | 0.43 | 2.68 | 0.01 | 1.18 | 2.92 |
| ***BMI For Age (BA)*** | | | | | | |
| z-score_BA_ $\leq$ –3.0 | 1.49 | 0.83 | 0.72 | 0.47 | 0.50 | 4.46 |
| z-score_BA_ > –3.0 and $\leq$ –2.0 | 1.21 | 0.29 | 0.80 | 0.43 | 0.75 | 1.95 |
| z-score_BA_ > –2.0 and < 1.0 | *Reference* | | | | | |
| z-score_BA_ $\geq$ 1.0 and < 2.0 | 0.85 | 0.09 | -1.63 | 0.10 | 0.69 | 1.03 |
| z-score_BA_ $\geq$ 2.0 and < 3.0 | 0.91 | 0.11 | -0.82 | 0.41 | 0.72 | 1.15 |
| z-score_BA_ $\geq$ 3.0 | 0.72 | 0.13 | -1.87 | 0.06 | 0.50 | 1.02 |
| ***Height For Age (HA)*** | | | | | | |
| z-score_HA_ $\leq$ –3.0 | 0.92 | 0.13 | -0.60 | 0.55 | 0.70 | 1.21 |
| z-score_HA_ > –3.0 and $\leq$ –2.0 | 1.18 | 0.14 | 1.44 | 0.15 | 0.94 | 1.49 |
| z-score_HA_ > –2.0 and < 3.0 | *Reference* | | | | | |
| z-score_HA_ $\geq$ 3.0 | 0.27 | 0.11 | -3.33 | 0.00 | 0.13 | 0.58 |
| ***Dialysis Status*** | | | | | | |
| On Dialysis | 3.37 | 0.29 | 13.94 | 0.00 | 2.84 | 3.99 |
| Off Dialysis | *Reference* | | | | | |
| ***Race/Ethnicity*** | | | | | | |
| White | *Reference* | | | | | |
| African American | 1.06 | 0.11 | 0.61 | 0.55 | 0.87 | 1.29 |
| Hispanic | 0.99 | 0.09 | -0.08 | 0.94 | 0.84 | 1.18 |
| Asian | 0.66 | 0.13 | -2.18 | 0.03 | 0.46 | 0.96 |
| Native American | 0.67 | 0.25 | -1.09 | 0.28 | 0.32 | 1.39 |
| Native Pacific Islander | 0.64 | 0.32 | -0.90 | 0.37 | 0.24 | 1.70 |
| Multiracial | 1.41 | 0.34 | 1.40 | 0.16 | 0.87 | 2.26 |
| ***cPRA values*** | | | | | | |
| cPRA = 0 | *Reference* | | | | | |
| cPRA > 0 and < 50 | 1.09 | 0.19 | 0.50 | 0.62 | 0.78 | 1.52 |
| cPRA $\geq$ 50 | 1.28 | 0.39 | 0.82 | 0.413 | 0.71 | 2.33 |
| ***Weight (kgs)*** | | | | | | |
| $\geq$ 0 and < 10 kgs | 0.40 | 0.10 | -3.83 | 0.00 | 0.25 | 0.64 |
| $\geq$ 10 and < 20 kgs | 0.82 | 0.08 | -2.03 | 0.04 | 0.67 | 0.99 |
| $\geq$ 20 and < 30 kgs | *Reference* | | | | | |
| $\geq$ 30 and < 40 kgs | 1.20 | 0.14 | 1.53 | 0.13 | 0.95 | 1.52 |
| $\geq$ 40 and < 50 kgs | 1.17 | 0.13 | 1.38 | 0.17 | 0.94 | 1.45 |
| $\geq$ 50 kgs | 0.98 | 0.08 | -0.20 | 0.84 | 0.83 | 1.16 |
| ***Kidney Disease Etiology*** | | | | | | |
| Acquired Defect | 0.42 | 0.25 | -1.48 | 0.14 | 0.14 | 1.32 |
| Amyloidosis | 1 | ---- | ---- | ---- | ---- | ---- |
| BK Virus Nephropathy | 1.28 | 1.81 | 0.17 | 0.86 | 0.08 | 20.45 |
| Cancer-Associated Nephropathy | 0.89 | 0.44 | -0.23 | 0.82 | 0.34 | 2.35 |
| Diabetes Mellitus I, II | 3.84 | 4.43 | 1.16 | 0.24 | 0.40 | 36.96 |
| Drug Induced Nephropathy | 0.18 | 0.19 | -1.59 | 0.11 | 0.02 | 1.48 |
| Heart Failure/Hypertension | 2.39 | 1.12 | 1.85 | 0.06 | 0.95 | 6.01 |
| HIV-Associated Nephropathy | 1 | ---- | ---- | ---- | ---- | ---- |
| Hypoplasia/Aplasia/Dysplasia | 0.82 | 0.07 | -2.49 | 0.01 | 0.70 | 0.96 |
| Immune Complex-Associated Nephropathy | 1.24 | 0.32 | 0.84 | 0.40 | 0.75 | 2.07 |
| Ischemic/Septic Nephropathy | 1.05 | 0.19 | 0.27 | 0.79 | 0.74 | 1.49 |
| MELAS | 1 | ---- | ---- | ---- | ---- | ---- |
| Metabolic Pathway Disorders | 0.32 | 0.25 | -1.45 | 0.15 | 0.07 | 1.50 |
| Nephritic/Nephrotic Syndromes | 1.43 | 0.14 | 3.53 | 0.00 | 1.17 | 1.74 |
| Nephrocalcinosis | 0.85 | 0.55 | -0.25 | 0.80 | 0.24 | 3.02 |
| Neurogenic Bladder | 1.08 | 0.45 | 0.19 | 0.85 | 0.48 | 2.42 |
| Prematurity-Associated Nephropathy | 1 | ---- | ---- | ---- | ---- | ---- |
| SLE | 0.54 | 0.14 | -2.32 | 0.02 | 0.32 | 0.91 |
| Tubular Dysfunction | 0.25 | 0.20 | -1.77 | 0.08 | 0.06 | 1.16 |
| Unspecified Autoimmune Disease | 1 | ---- | ---- | ---- | ---- | ---- |
| Unspecified Familial Kidney Disease | 1.71 | 1.30 | 0.70 | 0.49 | 0.38 | 7.64 |
| Unspecified Glomerulonephritis | 1.51 | 0.43 | 1.44 | 0.15 | 0.86 | 2.65 |
| Unspecified Interstitial Nephritis | 0.32 | 0.25 | -1.45 | 0.15 | 0.07 | 1.50 |
| Vasculidites | 1.64 | 0.33 | 2.47 | 0.01 | 1.11 | 2.42 |
| Unknown Etiology | *Reference* | | | | | |
| ***Insurance Type*** | | | | | | |
| Private Insurance | 0.92 | 0.08 | -0.91 | 0.36 | 0.78 | 1.10 |
| Public Insurance | 1.28 | 0.11 | 2.97 | 0.00 | 1.09 | 1.51 |
| Other Payment Source | *Reference* | | | | | |
| ***OPTN Region*** | | | | | | |
| Region 1 | 1.04 | 0.22 | 0.27 | 0.79 | 0.70 | 1.60 |
| Region 2 | 0.73 | 0.10 | -2.33 | 0.02 | 0.55 | 0.95 |
| Region 3 | 1.60 | 0.19 | 3.98 | 0.00 | 1.27 | 2.02 |
| Region 4 | 1.93 | 0.24 | 5.24 | 0.00 | 1.51 | 2.46 |
| Region 5 | 0.51 | 0.05 | -6.48 | 0.00 | 0.41 | 0.62 |
| Region 6 | 0.59 | 0.12 | -2.60 | 0.01 | 0.40 | 0.88 |
| Region 7 | 0.73 | 0.13 | -1.84 | 0.07 | 0.52 | 1.02 |
| Region 8 | 1.68 | 0.26 | 3.31 | 0.00 | 1.24 | 2.29 |
| Region 9 | 0.49 | 0.08 | -4.14 | 0.00 | 0.35 | 0.68 |
| Region 10 | 1.86 | 0.30 | 3.85 | 0.00 | 1.36 | 2.55 |
| Region 11 | 1.28 | 0.18 | 1.73 | 0.08 | 0.97 | 1.70 |

**SM Table 3 Univariable logistic regression.** This table displays factors that were significant in the univariable analysis, as well as the reference group for all variables where applicable. Age shows a bimodal distribution of significance with children less than one year old and children between 10-14 years being independently associated with either decreased or increased likelihood to transplantation. Notably, dialysis status is highly significant with a very large odds ratio of 3.37. Multiple regions are also significant, particularly region 9 which is associated with a 51% decrease in likelihood to transplantation within one year of listing. Blood groups B and AB, lower weight values, kidney hypoplasia/aplasia/dysplasia, nephritic/nephrotic syndromes, SLE, vasculitis, and public insurance are also factors significant in the univariable analysis. BMI-for-age, race/ethnicity (except Asian), and cPRA values are not significant.

| **Variable** | **Odds Ratio** | **Standard Error** | **z-score** | **P-value** | **95% Confidence Interval** | |
| --- | --- | --- | --- | --- | --- | --- |
| ***Age*** | | | | | | |
| $\leq$ 1 year | 0.87 | 0.23 | -0.52 | 0.60 | 0.52 | 1.46 |
| > 10 and $\leq$ 14 years | 1.08 | 0.12 | 0.75 | 0.45 | 0.88 | 1.34 |
| ***Blood Group*** | | | | | | |
| Group B | 0.65 | 0.09 | -3.19 | 0.00 | 0.49 | 0.84 |
| Group AB | 2.07 | 0.53 | 2.84 | 0.00 | 1.25 | 3.40 |
| ***Height For Age (HA)*** | | | | | | |
| z-score_HA_ $\geq$ 3.0 | 0.33 | 0.20 | -1.84 | 0.07 | 0.19 | 1.05 |
| ***Dialysis Status*** |  |  |  |  |  |  |
| On Dialysis | 3.67 | 0.36 | 13.26 | 0.00 | 3.03 | 4.45 |
| ***Race/Ethnicity*** | | | | | | |
| Asian | 0.81 | 0.17 | -1.03 | 0.30 | 0.54 | 1.21 |
| ***Weight (kgs)*** | | | | | | |
| $\geq$ 0 and < 10 kgs | 0.43 | 0.14 | -2.59 | 0.01 | 0.23 | 0.82 |
| $\geq$ 10 and < 20 kgs | 0.59 | 0.07 | -4.21 | 0.00 | 0.47 | 0.76 |
| ***Kidney Disease Etiology*** | | | | | | |
| Hypoplasia/Aplasia/Dysplasia | 1.14 | 0.13 | 1.16 | 0.25 | 0.91 | 1.42 |
| Nephritic/Nephrotic Syndromes | 1.17 | 0.16 | 1.16 | 0.25 | 0.90 | 1.52 |
| SLE | 0.38 | 0.11 | -3.25 | 0.00 | 0.21 | 0.68 |
| Vasculitis | 1.22 | 0.28 | 0.86 | 0.39 | 0.78 | 1.90 |
| ***Insurance Type*** | | | | | | |
| Public Insurance | 1.01 | 0.10 | 0.12 | 0.91 | 0.84 | 1.22 |
| ***OPTN Region*** | | | | | | |
| Region 2 | 0.73 | 0.12 | -1.85 | 0.07 | 0.52 | 1.02 |
| Region 3 | 1.29 | 0.20 | 1.67 | 0.10 | 0.96 | 1.75 |
| Region 4 | 1.35 | 0.22 | 1.87 | 0.06 | 0.99 | 1.85 |
| Region 5 | 0.54 | 0.08 | -4.41 | 0.00 | 0.41 | 0.71 |
| Region 6 | 0.46 | 0.11 | -3.34 | 0.00 | 0.30 | 0.73 |
| Region 8 | 1.45 | 0.27 | 1.97 | 0.05 | 1.00 | 2.10 |
| Region 9 | 0.58 | 0.12 | -2.67 | 0.01 | 0.39 | 0.87 |
| Region 10 | 1.64 | 0.32 | 2.59 | 0.01 | 1.13 | 2.40 |

**SM Table 4 Multivariable logistic regression results.** Multivariable logistic regression results. This table displays factors that were significant in the multivariable analysis. Blood groups B and AB, dialysis status, weight between 10 and 20 kg, SLE, and regions 5 and 6 were the most significant factors from this analysis. Dialysis status, SLE, and regions 5 and 6 had the most clinically significant odds ratios. Patients on dialysis were over three times more likely to receive a transplant within one year of listing while patients who had SLE had a 62% decreased likelihood for the same event. Patients located in regions 5 or 6 had 46% and 54% decreased likelihood, respectively.
